# The *Metarhizium anisopliae* Toxin, Destruxin A, Interacts with the SEC23A and TEME214 Proteins of *Bombyx mori*

**DOI:** 10.3390/jof7060460

**Published:** 2021-06-08

**Authors:** Fei Yin, Miaomiao Xiao, Alexander Berestetskiy, Qiongbo Hu

**Affiliations:** 1 Key Laboratory of Bio-Pesticide Innovation and Application of Guangdong Province, College of Plant Protection, South China Agricultural University, Guangzhou 510642, China; yinfei@gdaas.cn (F.Y.); xiaomiaomiao@stu.scau.edu.cn (M.X.); 2 Institute of Plant Protection, Guangdong Academy of Agricultural Sciences and Guangdong Provincial Key Laboratory of High Technology for Plant Protection, Guangzhou 510640, China; 3 Department of Phytotoxicology and Biotechnology, All-Russian Institute of Plant Protection, Shosse Podbelskogo, 3 Pushkin, 196608 Saint-Petersburg, Russia; aberestetskiy@vizr.spb.ru

**Keywords:** destruxin A, interaction, TEME214, SEC23, *Bombyx mori*

## Abstract

Destruxin A (DA), a mycotoxin isolated from the entomopathogenic fungus *Metarhizium anisopliae*, has good insecticidal and immune-inhibitory activity, but the action mechanism has not yet been elucidated. In order to identify the DA-binding proteins, we conducted drug affinity responsive target stability (DARTS) experiments, which indicated that the silkworm’s (*Bombyx mori*) transmembrane protein 214 (BmTEME214) and protein transport protein SEC23A isoform X2 (BmSEC23) are the potential DA-binding proteins. The current research was focused on validation of the interaction between DA and these two proteins via bio-layer interferometry (BLI) in vitro, insect two-hybrid (I2H) in Sf9 cells, and RNAi in the insect. The results of the BLI tests showed that DA has strong affinity to bind BmTEME214 and BmSEC23 proteins with a K_D_ value of 0.286 and 0.291 µM, respectively. In the I2H experiments, DA inhibited (at 0.02 µg/mL) and activated (at 0.002–0.0002 µg/mL) the protein interactions of BmSEC23–BmSEC13, but it only inhibited the BmTMEM214–BmSEC13L interaction. Furthermore, in the RNAi tests, an apparent increase in the silkworm’s mortality was recorded in the joint treatment of DA with dsBmSEC23 or dsBmTMEM214 when compared with the single treatment of DA (1.5 µg/g body), 40 µg/g body dsBmSEC23, or dsBmTMEM214. This research confirmed that BmSEC23 and BmTMEM214 are the DA-binding proteins and provided new insights to understand the action mechanism of DA.

## 1. Introduction

*Metarhizium anisopliae* is a well-known entomopathogenic fungus and plays an important role in pest biocontrol. This fungus has been used as a mycoinsecticide for many years worldwide. To date, there are 16 pesticides of *M. anisopliae* registered in mainland China (China Pesticide Information Network. Available online: http://www.chinapesticide.org.cn/hysj/index.jhtml, Accessed on 7 April 2021), while numerous similar products are found in the USA, European Union, Russia, and other countries [1,2,3]. However, these mycoinsecticides often show slow and unstable insecticidal effects, which limit their application. The defects of mycoinsecticides may result from the lack of sufficient knowledge of the insecticidal mechanisms of fungal pathogens [4]. Researching the mycotoxins produced by *M. anisopliae* can improve our understanding of the pathogenic mechanisms of the fungus, thereby promoting its application.

*M. anisopliae* produces destruxin, a cyclic hexadepsipeptide with multiple bioactivities, such as insecticidal, phytotoxic, antitumor, and antivirus activities [5]. Destruxin A (DA), the most common and active analogue of destruxins, is the key pathogenic factor of *M. anisopliae* against host insects [6]. Compared with the existing nerve poison type of pesticides, such as organophosphates, carbamates, pyrethroids, and diamides, DA has a distinct insecticidal activity through the inhibition of the host’s innate immunity, including depressing the production of antimicrobial peptides and damaging the hemocytes [7,8]. Therefore, DA is considered as a lead of novel insecticide [9]. However, its action mechanism has not yet been clearly elucidated.

In order to identify DA-binding proteins, we conducted experiments of drug affinity responsive target stability (DARTS) [10] in the hemolymph and Bm12 cells of the silkworm, *Bombyx mori*. Dozens of suspected DA-binding proteins were screened out, among them, transmembrane protein 214 (TEME214) and protein transport protein SEC23A isoform X2 (SEC23A) were frequently detected. These two proteins are located in the endoplasmic reticulum(ER). The humanTEME214 is known to play an important role in ER stress-induced apoptosis [11], while SEC23A is the component of the coat protein complex II (COPII), which promotes the formation of transport vesicles from ER and transports the cargo molecules to the Golgi complex [12,13]. 

Interestingly, DA, as well as endoplasmic reticulum stress, can induce apoptosis [5,14,15]. It is important to elucidate the interaction relations of DA with the two ER proteins. Therefore, in this study, we employ the technologies of bio-layer interferometry (BLI), insect two-hybrid (I2H), and RNAi to confirm that the silkworm’s TEME214 and SEC23A are DA-binding proteins. The results are reported below. 

## 2. Materials and Methods

### 2.1. Destruxin A (DA)

DA was isolated and purified from the *M. anisopliae* strain MaQ10 in our laboratory [16]. It was dissolved by dimethyl sulfoxide (DMSO, Sigma-Aldrich, Darmstadt, Germany) into a stock of 10,000 mg/L for short-term storage at −80 °C. 

### 2.2. Bio-Layer Interferometry (BLI) 

The two proteins, BmTEME214 and BmSEC23, were expressed by *Escherichia coli* with His-tag and purified by nickel affinity chromatography [17]. The affinity analysis was performed on a ForteBio OctetQK System (K2, Pall Fortebio Corp, Menlo Park, CA, USA). The protein samples were coupled with a biosensor for immobilization. Serial dilutions (7.81, 15.62, 31.25, 62.5, and 125 µmol/L) of DA were used for treatments. PBST buffer (0.05% Tween20, 5% DMSO) was used for the running and dilution buffer. The working procedure was baseline for 60 s, association for 60 s, and dissociation for 60 s. Finally, the data analysis software (9.0, Pall Fortebio Corp, Menlo Park, CA, USA) was used to evaluate the affinity constants.

### 2.3. Insect Two-Hybrid (I2H)

The I2H tests were conducted in the *Spodoptera frugiperda* 9 (Sf9) cell line by referring to Wang et al. [18]. In brief, Sf9 cells were cultured in SFX culture medium (Hyclone, Pittsburgh, MA, USA) with 5% fetal bovine serum (Gibco, Waltham, MA, USA) for 2–4 days at 27 °C. The logarithmic phase cells were used for the I2Hexperiment. By using the Gateway system, the sequences of *BmTEME214* and *BmSEC23* were cloned into the I2H vector pIE-AD, while their interaction proteins genes, *BmSEC13L* and *BmSEC13* (Table 1), were transferred into the I2H vector pIE-DBD. Then, the destination vectors pIE-AD-BmTEME214 and pIE-DBD-BmSEC13L with the reporting gene luciferase vector were co-transfected into the Sf9 cells, while pIE-AD-BmSEC23 and pIE-DBD-BmSEC13 with the luciferase vector were co-transfected into SF9 cells. After 24 h of incubation at 27 °C, the DA solution was added in the cell cultures at final concentrations of 0.0002, 0.002, 0.02, 0.2, and 2 µg/mL. After 24 h, the luciferase activities in the cell extracts were determined using a Luciferase Reporter Assay System (Promega, Beijing, China) and Synergy™ H1 (BioTek, Winooski, VT, USA). The experiments were replicated three times. The control group was only treated with DMSO. Statistical analysis was carried out using SPSS software (IBM, Armonk, NY, USA). The results are presented by mean ± SD (standard deviation). Statistical significance was tested using a one-way ANOVA; the differences among the means were compared by Tukey’s HSD.

### 2.4. RNAi Bioassay

The dsRNA of *BmTMEM214* and *BmSEC23* genes were synthetized according to the instructions of the T7 RioMAX^TM^ Express RNAi System in vitro. After 6 h of starvation, second instar larvae of silkworms were fed with mulberry leaves coated with dsRNA (40 µg/g body). After 24 h of dsRNA treatment, the silkworms were injected with DA at a dose of 1.5 µg/g body. The tests were repeated three times, and 10 silkworms were used for each replication. The DMSO-only treatment group (DMSO) (CK) was used as control. The qPCR was used to survey the gene expression levels after treatment. The reaction system consists of 1 μL of cDNA of reverse transcription, 1 μL of upstream/downstream primers, 10 μL of SYBR Premix Ex Taq^TM^ (Bioscience, Shanghai, China), and 7 μL of ddH_2_O. The qPCR reactive program was subjected to 39 cycles at 95 °C for 10 s, 60 °C for 10 s, 72 °C for 30 s, then 95 °C for 10 s, and 65−95 °C for 5 s. The silkworm *GAPDH* (glyceraldehyde-3-phosphate dehydrogenase) was taken as the reference gene. The 2^−^^Δ^^ΔCt^ method was used to analyze the qPCR data in gene expressions of RNAi treatments. The relative expression of the target genes in the experimental treatment group (Q) was Q = 2^−ΔΔCt^ [19]. The survival rates of silkworm were evaluated daily within 12 days post-treatment.

## 3. Results

### 3.1. BLI of DA with BmTEME214 and BmSEC23

**Figure 1:**
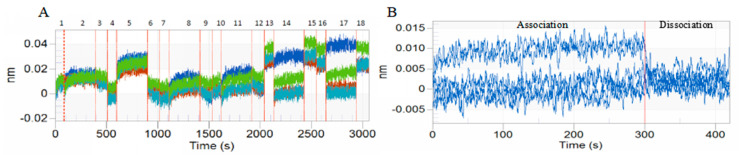
Kinetic curves and align analysis of the interaction between DA and BmTMEM214 in BLI tests. (**A**) Interaction curve between BmTMEM214 and DA. **–**SSA sensor of solidified BmTMEM214 with serial dose of DA. **–**SSA sensor of solidified BmTMEM214 reference buffer. **–**SSA sensor of non-solidified protein and serial dose of DA. **–**SSA sensor of non-solidified protein and reference buffer. (**B**) Align X diagram of association and disassociation in the interaction between DA and BmTMEM214.

**Figure 2:**
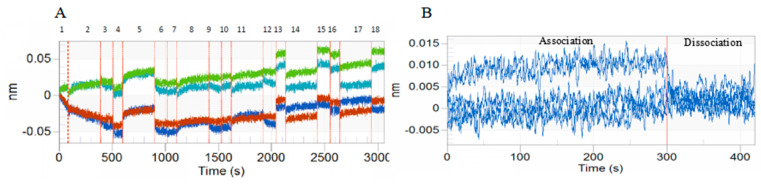
Kinetic curves and align analysis of the interaction between DA and BmSEC23in BLI tests. (**A**) Interaction curve between BmSEC23 and DA. **–**SSA sensor of solidified BmSEC23 and serial dose of DA. **–**SSA sensor of solidified BmSEC23and reference buffer. **–**SSA sensor of non-solidified protein and serial dose of DA. **–**SSA sensor of non-solidified protein and reference buffer. (**B**) Align X diagram of association and disassociation in the interaction between DA and BmSEC23.

The results of the BLI tests indicated that DA has strong affinity with BmTEME214 (Figure 1) and BmSEC23 (Figure 2). The affinity constant K_D_ values of DA with BmTEME214 and BmSEC23 were 0.286 and 0.291 µM, respectively (Table 2).

### 3.2. I2H of DA with BmTMEM214 and BmSEC23

**Figure 3:**
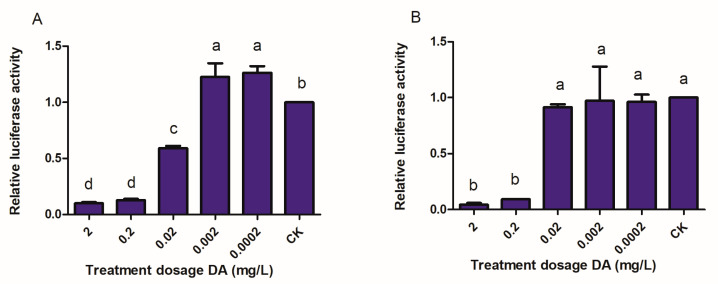
Effects of DA on the interactions of BmTMEM214–BmSEC13L (**A**) and BmSEC23–BmSEC13 (**B**) by I2H tests. The different letters on the columns indicate the significant difference (*p*< 0.05) by Tukey’s HSD test. CK: DMSO-only treatment group.

The results showed that DA considerably influences the interactions of BmTMEM214–BmSEC13L and BmSEC23–BmSEC13 (Figure 3). For the BmTMEM214–BmSEC13L interaction, DA at higher dose (0.02 µg/mL) inhibited the interaction, while at lower doses (0.002–0.0002 µg/mL), it activated the same interaction, because the activities of luciferase decreased by ~40%andincreased by ~20% were found in higher dose and lower dose DA treatments, respectively (Figure 3A). However, a DA dose-dependent relative luminescent value decrease was recorded in BmSEC23–BmSEC13, where only at the dose ≥0.2 µg/mL did DA significantly inhibit the interaction between the proteins (Figure 3B). 

### 3.3. Toxicity of DA against Silkworm under RNAi 

**Figure 4:**
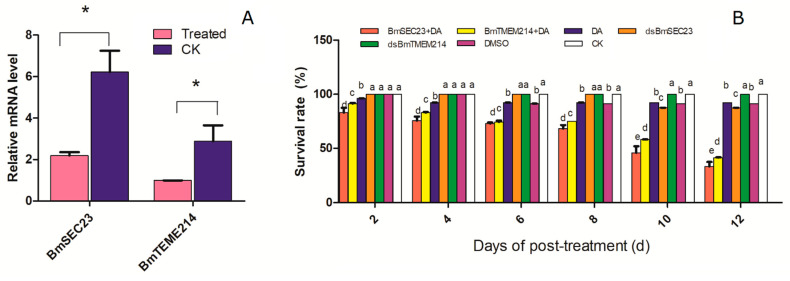
The effect of gene silencing on DA toxicity against the silkworm in RNAi tests. (**A**) The relative expression of BmSEC13 and BmTMEM214 in the silkworm after feeding dsRNA. (**B**) The mortality of the silkworm after treatments with DA 1.5 µg/g body, dsBmSEC23, and dsBmTMEM214 40 µg/g body. DMSO: DMSO injection only. CK: normal feeding without any treatment. The star (*) in (**A**) indicates the significant difference (*p* < 0.05) by *t*-test. The letters on the column in (**B**) indicate the significant difference (*p* < 0.05) by Tukey’s HSD.

It was indicated that the relative gene expressions of *BmSEC13* and *BmTMEM214* decreased significantly by >65% after dsRNA treatments (40 µg/g body), which suggested the good efficiency of RNAi (Figure 4A). In bioassay tests, a single treatment of dsRNA or DA had >85% survival rates on 2–12 days post-treatment, and these results are similar with those of the DMSO and normal feeding (CK) groups (Figure 4B). However, the combination treatments of dsRNA and DA had survival rates of only 30–80%. It was suggested that the joint treatment significantly increased silkworm mortality. Clearly, the increase in DA toxicity is caused by the decrease in BmTEME214 or BmSEC23 by RNAi. The results provide new evidence for the interactions of DA with the two proteins.

## 4. Discussion

The interactions between DA and BmTEME214 and BmSEC23 proteins were proved by BLI, I2H, and RNAi in this study. The BLI data show that DA binds to the two proteins with similar affinity levels (K_D_ values of 0.286 and 0.291 µM, respectively). However, the K_D_ values are apparently lower than the 10 µM level of DA with BmHSCP [17], BmTudor-sn [18], and BmPPI [20], which suggests that the affinities of DA with BmTEME214 and BmSEC23 are of a higher level.

The interactions of DA with the two proteins were further validated by I2H tests. However, the I2H results indicate that DA binds to BmTMEM214–BmSEC13L much more successfully than to BmSEC23–BmSEC13, because DA, even at dose of 0.0002 mg/L, affects BmTMEM214–BmSEC13L interaction, while at only ≥0.2 mg/L, it suppresses BmSEC23–BmSEC13 interaction. It might be that BmSEC23–BmSEC13 has higher interaction that leads to a decrease in DA binding to BmSEC23.

Furthermore, the RNAi experiments in silkworm larvae provide indirect evidence to of the interactions between DA and BmTEME214 and BmSEC23. The joint treatments of DA with RNAi significantly increased the mortality of the silkworm, which is probably related to quantity decreases in the two proteins caused by partial knock-down of genes by dsRNA treatments, leading to less free proteins existing in the cell for DA binding.

Interestingly, the I2H results indicate that DA inhibits the BmTMEM214–BmSEC13L interaction at a higher dose (0.02–2 mg/L), while at lower concentrations (0.002–0.0002 mg/L), the toxin activates the interaction. TMEM214 has been scarcely studied. Through Uniprot blast, BmTEME214 (XP_004933467.1) was found to have 30% similarity with the human transmembrane protein 214 (HsTMEM214) (UniProtKB, Q6NUQ4), the latter playing an important role in endoplasmic reticulum (ER) stress-induced apoptosis by acting as an anchor for recruitment of procaspase 4 to the ER [11]. There are no experimental reports about proteins interacting with BmTEME214, so we selected BmSEC13L (NP_001040420.1) as a candidate partner according to the prediction of String. Consequently, we demonstrated the BmTEME214–BmSEC13L interaction. Although belongs to the SEC13 family due to WD repeat domain, BmSEC13L is less similar to SEC13. On the contrary, BmSEC13L is more homologous to nucleoporin Seh1 (53.5% identity with Q7K2X8, the nucleoporin Seh1 of *Drosophila melanogaster*, and 59.3% with Q96EE3, the nucleoporin Seh1 of humans). Seh1 is a structural protein and a component of the nuclear pore complex, which is involved in maintaining the localization of another nucleoporin mTOR (mechanistic target of rapamycin) to the nuclear envelope of early meiotic female germline cells [21,22]. The mTOR is an atypical serine/threonine kinase that is the center of the mTOR signal pathway through two distinct complexes [23]. The mTOR complex 1 (*mTORC1*) is a master growth regulator that senses and integrates diverse nutritional and environmental cues, including growth factors, energy levels, cellular stress, and amino acids. It couples these signals to the promotion of cellular growth by phosphorylating substrates that potentiate anabolic processes, such as mRNA translation and lipid synthesis, and it limits catabolic processes such as autophagy. The mTOR complex 2 (*mTORC2*) promotes cellular survival, regulates cytoskeletal dynamics, and controls ion transport. The effects of DA on BmTMEM214–BmSEC13L interaction naturally influence the mTOR signal pathway. However, the function and significance of BmTEME214–BmSEC13L interaction require further research. Furthermore, BmTMEM214 warrants in-depth investigation, because BmTMEM214 and HsTMEM214 exhibit limited homology. 

SEC23A has good conservation: the silkworm’s SEC23A (XP_012553105.1) shares 75% identity with the homologues of mice (Q01405) and humans (Q15436). The SEC23A is a component of the coat protein complex II (COPII), and it promotes the formation of transport vesicles from the endoplasmic reticulum (ER). The COPII is involved in the physical deformation of the ER into vesicles and the selection of cargo molecules for their transport to the Golgi complex [12,13]. The COPII coat consists of the four heterotetramers (SEC13/31 and SEC23/24) and the GTP binding protein Sar1. The hinge region formed by the four heterotetramers can direct cage expansion to accommodate cargo of various sizes [24,25]. Furthermore, the binding of SEC23 induces a conformational change in SEC13/31, resulting in a more extended conformation [26]. Similarly, SEC13 is conserved, and BmSEC13 (XP_004923349.1) has 65% identity with the homologues of mice (Q9D1M0) and humans (P55735), which have been thoroughly investigated. Therefore, based on this study, we can deduce that DA binds to SEC23 and affects SEC23–SEC13 interaction, subsequently deterring the formation and assembly of COPII vesicles. Eventually, the homeostasis of organelles is broken, and the cellular function is destroyed. Further research to determine whether DA activates or inhibits the binding of SEC23 to SEC13/31 is warranted.

In conclusion, in this research, we confirmed that the silkworm’s BmTEME214 and BmSEC23 are the DA-binding proteins. Furthermore, DA affects the protein–protein interaction in the cases of BmTEME214–BmSEC13L and BmSEC23–BmSEC13. This study provides new insights that help us further our understanding of the molecular mechanisms of DA action in insects.

## Figures and Tables

**Table 1:** Information of proteins used in I2H tests.

Target Protein			Interacting Protein			Interaction Score (String)
Name	ID (NCBI)	Description	Name	ID (NCBI)	Description	
BmTMEM214	XP_004933467.1	transmembrane protein 214	BmSEC13L	NP_001040420.1	SEC13-like protein	0.578
BmSEC23	XP_012553105.1	protein transport protein SEC23A isoform X2	BmSEC13	XP_004923349.1	Protein SEC13 homolog	0.995

**Table 2:** Affinity data of DA with the two proteins determined by BLI.

Protein	K_D_ (µM) ^a^	K_ON_ (1/µMs) ^b^	K_OFF_ (1/s) ^c^	Full R^2^
BmTMEM214	0.286	0.184	0.0528	0.754
BmSEC23	0.291	1.50	0.436	0.935

**^a^**: affinity constant K_D_; ^b^: association rate constant K_ON_; ^c^: dissociation rate constant K_OFF_.

## Data Availability

All relevant data are within the manuscript.
